# Young people’s and stakeholders’ perspectives of adolescent sexual risk behavior in Kilifi County, Kenya: A qualitative study

**DOI:** 10.1177/1359105317736783

**Published:** 2017-10-27

**Authors:** Derrick Ssewanyana, Patrick N Mwangala, Vicki Marsh, Irene Jao, Anneloes van Baar, Charles R Newton, Amina Abubakar

**Affiliations:** 1 Kenya Medical Research Institute (KEMRI), Kenya; 2 Utrecht University, The Netherlands; 3 Pwani University, Kenya; 4 University of Oxford, UK

**Keywords:** adolescence, qualitative methods, risk factors, sexual behavior, youth

## Abstract

A lack of research exists around the most common forms of sexual risk behaviors among adolescents, including their underlying factors, in Sub-Saharan Africa. Using an Ecological Model of Adolescent Behavior, we explore the perceptions of 85 young people and 10 stakeholders on sexual risk behavior of adolescents in Kilifi County on the coast of Kenya. Our findings show that transactional sex, early sexual debut, coerced sex, and multiple sexual partnerships are prevalent. An urgent need exists to develop measures to counter sexual risk behaviors. The results contribute to understanding the range of risks and protective factors in differing contexts, tackling underlying issues at individual, family, local institutional, wider socio-economic, and political levels.

## Background

Sexual risk behavior (SRB) and forms of sexual exploitation faced by adolescents in Sub-Saharan Africa (SSA) need to be addressed, since SRB, such as early sexual debut, multiple sexual partnerships, unprotected sex, intimate partner violence, and sexual exploitation through early marriage and transactional sex, have been extensively documented among adolescents within this region (Bankole et al., 2007; Idele et al., 2014; Wamoyi et al., 2015). Given this state, it is not surprising that SSA accounts for almost two-thirds of the 300,000 new HIV infections among adolescents between 15 and 19 years globally (Idele et al., 2014). Likewise, the adolescent birth rate in SSA was the highest globally (117 per 1000 girls aged 15–19 years) for the period from 1991 to 2010 (United Nations Population Fund (UNFPA), 2013).

In SSA, Kenya is one of the eight countries that registered the highest rates of new HIV infections in 2015, and sexual intercourse remains the predominant route of HIV transmission (The Joint United Nations Programme on HIV/AIDS (UNAIDS), 2016). Only 35 percent of sexually active Kenyan adolescents report having used any form of contraception (Obare et al., 2016), early sexual debut and cross-generational sex are prevalent (Luke, 2005; Nzioka, 2001), and 25 percent of the Kenyan adults report having experienced some form of sexual violence before the age of 18 years (United Nations International Children’s Emergency Fund (UNICEF) et al., 2012). Moreover, disparities exist such that adolescents living in the coastal region of Kenya face a disproportionately poorer state of adolescent sexual and reproductive health (ASRH) than those from most regions within the country (Kenya National Bureau of Statistics (KNBS) and ICF International, 2014). Teenage pregnancy rate is highest (21%) at the Kenyan coast, and the unmet need for family planning among married adolescent girls (15–19 years) is 59 percent, which is twice more than the national prevalence of 23 percent (KNBS and ICF International, 2014). Furthermore, other concerns of sexual violence, sexual tourism, early marriages, and high school dropout rates are reported at the Kenyan coast (National AIDS Control Council (NAAC), 2016; National Crime Research Centre (NCRC), 2014), which emphasize the urgent need for intervention around adolescent SRB in this context. Adolescent SRB has been linked to consequences such as high teenage pregnancy (currently at 18% in Kenya) (Obare et al., 2016), unsafe abortions, school dropout, mental ill health, and sexually transmitted infections (Godia et al., 2014; Juma et al., 2014; UNICEF et al., 2012).

The assessment of different forms of SRB and their facilitating or contextual factors is a vital step toward designing effective strategies and interventions for SRB (Hanson et al., 2015; Wight et al., 2016). In the Kenyan context, just a few studies have comprehensively explored underlying factors for adolescents’ SRB. In western Kenya, socio-cultural factors such as funeral ceremonies, boy child preference, early marriage, and widow inheritance are among reported factors for SRB (Juma et al., 2014; Njue et al., 2009). Studies from Central (Caroline and Orpinas, 2008; Marston et al., 2013) and Eastern (Nzioka, 2001) regions of Kenya describe peer pressure, low risk perception, lack of parental supervision, lack of school attendance, family dysfunction, alcohol and drug use, delinquent behavior, and gender norms as some predisposing factors for adolescent SRB. Nonetheless, it is not clear whether unique factors explain the disproportionately poor state of ASRH at the Kenyan coast since there is a paucity of literature on drivers of adolescent SRB associated with this specific setting. This presents challenges in guiding intervention and policy development (NAAC, 2016). We therefore seek to engage adolescents and local community stakeholders to reflect on mechanisms by which SRB is generated and normalized within their context, as this avoids attributing SRB to merely broad and generic social norms (Hanson et al., 2015).

To contribute toward a clearer understanding of the prevalent adolescent SRB and their underlying factors, we conducted a qualitative study to understand young people’s and stakeholders’ perspectives on SRB among adolescents (10–19 years) in Kilifi County at the Kenyan coast. Specifically, we explored perceptions of (1) most common forms of SRB, (2) predisposing or risk factors for SRB, and (3) protective factors against SRB among adolescents. We draw on the perceptions of young people and a range of other community stakeholders to explore diversity and the convergence of viewpoints and factors underlying adolescent SRB behavior within this setting.

## Theoretical framework

We utilize the Ecological Model of Adolescent Behavior by Blum et al. (2001), which provides a logical account of underlying factors responsible for the distribution and occurrence of adolescent risk behavior. This model draws on ecological systems theory which posits that understanding human development requires the examination of multi-person systems of interaction and that this should take into consideration the numerous aspects of the environment beyond an immediate situation concerning the subject (Bronfenbrenner, 1977). The model by Blum and colleagues postulates that there are intricate mechanisms that underlie risk behavior and these involve interactions of multiple explanatory domains (Blum et al., 2001). This model proposes six major explanatory domains or sources of behavioral variation and each of these domains comprises risk factors and protective factors. These domains are as follows: (1) individual, (2) family, (3) peers, (4) school, (5) social environment, and (6) macro-environment (Blum et al., 2001). All the domains are presumed to have direct effects on adolescent risk behavior; however, there is interplay of factors within and across domains, which explains the various forms of risk and protection. Moreover, various domains of risk can also have indirect effects on risk behavior. The model presumes a bi-directionality between the risk factors and behavioral outcomes, that is, engaging in a risk behavior can also affect the various domains of risk factors. The protective factors are considered to moderate or counter-balance and thus mitigate the impacts of risk on adolescent behavior (Blum et al., 2001). This model suggests that family, peer, and school influences on the adolescent fall within a broader social environment which also ultimately lies within a macro-environment.

## Methods

### Study setting and participants

This qualitative study was conducted between August and November 2016 at the Kenya Medical Research Institute (KEMRI)-Wellcome Trust Research Programme (KWTRP), which has its main base within Kilifi County at the Kenyan coast. Data were collected within a well-defined health and demographic surveillance area, the Kilifi Health and Demographic Surveillance System (KHDSS). By 2011, the population of KHDSS was estimated at 270,000 residents, and 49 percent were less than 15 years (Scott et al., 2012). The residents mainly belong to the Mijikenda ethnic group, which is a Bantu group comprising nine sub-groups with a majority (45%) being Giriama. The main form of livelihood within this community is subsistence farming. Kilifi County is mainly served by 1 major hospital (KCH), 1 health center, and 12 dispensaries (Scott et al., 2012).

This study involved the following two groups of study participants:

1. Adolescents aged 10–19 years and young adults between 20 and 30 years who were Kilifi residents and serving as community representatives at KWTRP. The adolescents were sub-divided into three groups, including those who were school-going (both primary and secondary students), had “dropped out” of school, and were living with HIV and attending an HIV Comprehensive Care Clinic at Kilifi County Hospital (KCH).
2. Key informants, who were female and male adults working extensively with adolescents within Kilifi, for example, teachers, employees of community-based organizations (CBOs) addressing adolescent health challenges, community social workers, clinicians, and county government staff.

### Recruitment and eligibility

Recruitment into this study was through a snowballing process. Initial contact was made with a small number of researchers at the Centre for Geographic Medicine Research-Coast (CGMR-C) in Kilifi whose work involved adolescents. They recommended additional key informants who should be contacted for interviews, based on experience of working with adolescents living with HIV. This process was repeated with all new interviewees to continue to identify participants sharing relevant perspectives for the study. In the school setting, head teachers suggested a contact teacher (who in most cases was a guidance and counseling teacher) who served as a key informant.

At KWTRP, we were able to draw on an existing network of community representatives to identify participants for this study (Kamuya et al., 2013). Using an existing database of around 200 community representatives in the Community Engagement department at KWTRP, we purposively selected community members aiming to reflect diversity within a young adult population, taking account of residential area (peri-urban or rural settings), education status, sex, and religion.

For school-going adolescents, we purposively identified two primary and two secondary schools in the KHDSS area, as before aiming to explore diversity. Characteristics identified included the setting and type of schools, specifically, schools in peri-urban and rural settings, and day schools attended by both male and female students. For study purposes, we considered Kilifi town as a reference peri-urban setting and areas with limited social services and within 10 or more kilometers from Kilifi town as rural settings. In consultation with Kilifi County Education Office and researchers at the CGMR-C with ongoing work within the school setting, we identified the four schools. Upper-primary school students (i.e. from class 5 to class 8) and lower-secondary school students (i.e. from forms 1 and 2) were purposively recruited from class records with a specific consideration of sex, religion, and age. School dropout adolescents were recruited with assistance from the County department of Public Health’s community support staff. Recruitment of the school dropout adolescents was done by purposively selecting them from two community health catchment units with consideration of age, sex, and religious diversity. The adolescents living with HIV were recruited from the youth club at the comprehensive care and research clinic of KCH, and some were recruited directly from the community through home visits to families of HIV-infected adolescents by a worker from the KCH.

### Ethical consideration

All recruitment processes involved obtaining prior written informed consent. Consent was directly sought from participants aged 18 years and over. For those aged under 18 years, parents or legal caretakers provided consent. In addition, for participants between 13 and 18 years, the adolescent’s assent was also sought. Permission to involve schools in this study was obtained from the County director of education and head teachers at each school involved.

The ethical clearance for this study was granted by the Kenya Medical Research Institute Scientific and Ethics Review Unit (KEMRI/SERU/CGMR-C/0047/3263).

## Data collection

We conducted in-depth interviews lasting on average 60–90 minutes with each of the key informants at a time of their convenience and at their preferred venue. We conducted focus group discussions (FGDs) comprising between seven and nine participants each lasting between 75 and 120 minutes with the young adult community representatives and adolescents. For school-going adolescents, FGDs were conducted within the school and were sex disaggregated. Young adult community representatives, adolescents living with HIV, and those who had dropped out of school were invited to participate in FGDs at the KWTRP, in a private and quiet setting. Participants (young adults) and caretakers of adolescents who were invited to CGMR-C were given 300 Kenyan shillings (about US$3) to compensate for time spent, and transport expenses were reimbursed. All interviews and discussions were moderated by a trained Research Officer in English and/or Kiswahili based on the participants’ preference. Permission was asked from participants to take notes and to audio record the discussions and interviews conducted.

Both the qualitative interview guide and the FGD guide were developed with guidance from the World Health Organization and the Centers for Disease Control and Prevention documents (CDC, 2015; WHO, 2015) to cover a wide range of adolescent health risk behaviors such as SRB, behavior resulting in unintentional injuries and self-harm, alcohol use behavior, smoking, drug use, poor dietary, physical activity, and poor hygiene behaviors. For each form of behavior, respondents’ perceptions were explored using a general open-ended question, followed by additional probing, that asked them to explain the specific forms of risky behaviors which they perceive as commonly undertaken by adolescents aged 10–19 years in Kilifi. We only probed further on specific examples or context of SRB that were raised by the participants and thus all the perceptions documented in this article were spontaneous views from the participants. We also collected respondents’ demographic information, such as age, sex, education status, residence, position held/affiliation, and religious background.

## Data analysis

The audio-recorded interviews and discussions were transcribed verbatim by a professional team and later translated into English. Two of the authors (D.S. and P.N.M.) critically scrutinized the scripts and developed initial codes from a priori and emergent issues. The final round of coding was conducted in NVivo 11 software (QSR International Ltd, Southport, UK) by D.S. Through a series of meetings within the research team, the codes were discussed and consensus reached on how these should be brought together into themes with guidance from the Ecological Model of Adolescent Behavior (Blum et al., 2001). Charting was then done by D.S. using a case and theme–based approach in Microsoft Excel. This framework was reviewed, discussed, and agreed upon by the research team to inform the ongoing analysis and interpretation of the results.

## Results

A total of 11 FGDs were held, of which 8 were sex disaggregated and conducted among school-going adolescents. The rest were not sex disaggregated and involved adolescents living with HIV, those who dropped out from school, and young adult community representatives. Also, 10 in-depth key informant interviews were conducted with 4 employees of CBOs, 2 County hospital staff, 3 teachers responsible for guidance and counseling affairs, and 1 County government staff. The total number of participants in this study was 95, of whom 77.9 percent were Christians and the rest were Muslim (see Table 1).

**Table 1.** Socio-demographic characteristics of participants.

Participants	Total	Age range (median age)	Sex	Education level
Key informants	10	27–51 (31)	4 Males, 6 Females	University degree (5), post-secondary level (5)
Young adult community representatives	7	22–28 (25)	3 Males, 4 Females	College level (4), secondary level (2), primary level (1)
Primary school adolescents				
Peri-urban male students	7	10–14 (14)	Males	Classes 5–8
Peri-urban female students	7	10–13 (12)	Females	Classes 5–8
Rural male students	8	12–16 (13.5)	Males	Classes 5–8
Rural female students	8	13–16 (14.5)	Females	Classes 5–8
Secondary school adolescents				
Peri-urban male students	8	16–19 (17)	Males	Forms 1 and 2
Peri-urban female students	6	15–17 (16)	Females	Forms 1 and 2
Rural male students	9	16–18 (17)	Males	Forms 1 and 2
Rural female students	9	15–18 (16)	Females	Forms 1 and 2
Adolescents living with HIV	9	12–19 (13.5)	5 Males, 4 Females	Class 3–Form 1
School dropout adolescents	7	12–18 (14)	5 Males, 2 Females	Class 2–Form 2

## Prevalent forms of SRB

The perceived forms of SRB are summarized in Table 2. Many of the participants had a general opinion that many of these forms of SRB, including transactional sex, cross-generational sex, early sexual debut, coerced sex, and multiple sexual partnerships, are common within Kilifi. Overall, there was agreement about the high occurrence of transactional sex and that this occurs in both sexes. These sexual relationships were described as occurring outside marriage or commercial sex work, but structured by an assumption that sex will be exchanged for material benefit or status (Stoebenau et al., 2016). Participants mainly cited gifts exchanged for sex as including: school fees, transport to school, sanitary pads, money, food, footwear, and clothes. As one participant described,

Teenagers are getting sugar mummies who finance their lifestyles in return for the teenagers offering them sex. (Group 3, male student, rural secondary school)

**Table 2.** Perceived forms of SRB among adolescents in Kilifi.

Sexual risk behavior	Number of FGDs where SRB described (*N* = 11 FGDs)	Number of key informants describing SRB (*N* = 10 KIs)
Transactional sex (gifts for sex)	11	10
Cross generational sex	11	9
Forceful sexual intercourse (an adolescent is the victim)	11	9
Early sexual debut	10	8
Multiple sexual partnerships	9	6
Unprotected sexual intercourse	8	8
Males having sex with males	3	5
Forceful sexual intercourse (an adolescent is the perpetrator)	5	1
Prostitution	3	2
Other forms		
Sexual harassment	3	0
Sex under influence of drugs	1	0

SRB: sexual risk behavior; FGD: focus group discussion; KI: key informant.

Likewise, sexual violence, and forms of sexual exploitation were characterized by age and economic asymmetries, and “sugar daddy/mummy” situations were commonly talked about. Adolescent girls were considered most vulnerable to these injustices, as explained by a participant:

She was forced by the father to have sex … a lady can be abused by the father, they have no right in the local region here (referring to the girls). As long as they are poor, anyone who comes there can use them. (KI, medical social worker, male, 28 years)

Early sexual debut coupled with multiple sexual partnerships and unprotected sex were also described across a majority of the participant groups:

Boys here in school, in fact they are very frank. They can tell you, “Madam, if I move with this girl today (referring to sexual relationship), next week I do not have that girl, I will leave that girl for someone else, I will go to someone else” so they keep on moving. (KI, secondary school teacher, female, 32 years)

Notably, some perceived that the age of sexual debut could be as early as 10 years:

It is extremely shocking that you find 10 year old, 11 year old, 12 year olds, they are sexually active and they are moving around with boys. (KI, CBO director, female, 46 years)

## Risk and protective factors for SRB

**Figure 1.**
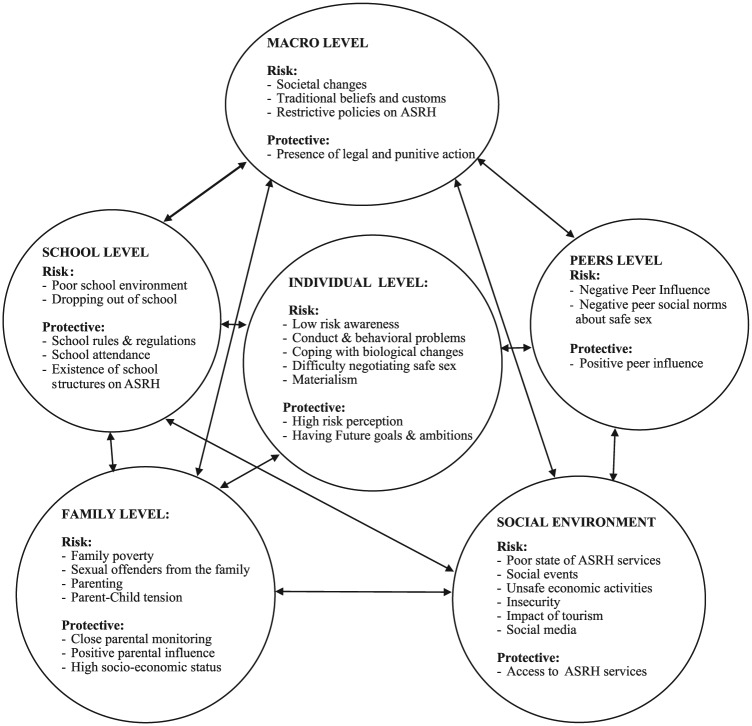
A flow diagram of young people’s and stakeholder’s perceived risk and protective factors for sexual risk behavior among adolescents living in Kilifi County.

Participants discussed a range of predisposing and protective factors for SRB, most factors predisposed to risk, and fewer protective influences were noted. Following the Ecological Model of Adolescent Behavior (Blum et al., 2001), a majority of the risk factors acted at a social environment level, followed by those functioning at individual- and family-level influences. In contrast, most protective factors described operated at school, social, and family levels (see Figure 1). These influences are described in the following paragraphs.

### Social environment factors

#### Health services

The current state of ASRH services was perceived as critical in both protecting from and in generating higher risks of SRB for adolescents in Kilifi. This was dependent on the extent to which the participants perceived accessibility, quality, and comprehensiveness of these services within the county. Where young people and key informants perceived ASRH services in Kilifi to be poor in quality and not adolescent-friendly, they expressed fears about seeking these services at government health centers. Some adolescents described that their peers chose to take condoms from health facilities at night when no one would notice. Poor prioritization of adolescent specific services by sexual health service planners and providers was also described as underpinning SRB. Furthermore, a lack of effective behavioral change communication methods and forums for girls to learn about contraception and safe sex was discussed:

It is like educating the youth about this issue (AIDS) has been neglected … many people here assume contraceptive matters are for those who already have children. (Group 11, male, community representative)

We talked to the girls and we said they need a professional counselor. And I think they also need some clips to show (referring to audio-visual ASRH messages), if they see unlike just hearing, I think they change their mind. (KI, secondary school teacher, female, 32 years)

However, accessibility to ASRH services was also discussed as a protective factor by both young people and stakeholders. During their discussions, a mix of ASRH services and their providers were identified by the participants. For example, adolescents mentioned private clinics, public health facilities, and peer educators as sources of services such as condoms, ASRH information, emergency contraceptives (e-pills), and long-term birth control methods. The key informants also mentioned similar examples in addition to teens’ club, condom dispensers in recreational places such as pubs and the chief’s office as sources of ASRH.

#### Social events

Particular social events, especially traditional ceremonies like parties at funerals and weddings, were often perceived as occasions during which many adolescents might either engage in SRB or face a risk of sexual exploitation. During such events, unprotected sex, sexual violence, cross-generational sex, and other behaviors were described as common, related to limited monitoring by parents and other authorities, and the use of drugs and alcohol:

… in that *matanga* (a local word referring to funeral ceremonies), there is free sex. … You see it is a chance for these adolescents to engage in sex. You don’t care about who your partner is because it is like a sex orgy. (KI, secondary school teacher, female, 51 years)

#### Economic activities

Engaging adolescent girls in potentially unsafe economic activities, particularly businesses selling alcohol was cited. Girls are asked by their parents to serve in such business which sometimes is the family’s main source of income or they find employment in other alcohol serving premises where they encounter various forms of sexual advances and harassment from male patrons:

Some women involve their girls in their business of selling *mnazi* (a local alcoholic brew) even overnight … as the young girls interact with the drinking customers they might be raped. (Group 3, males, rural secondary school)

But what the older women do now, they call young women to come around to help in serving; just as a way to attract the *wazee* (meaning older male patrons) … And they do not drink the beer, but the men there can get the girls. (KI, CBO employee, male, 27 years and KI, secondary school teacher, female, 51 years)

#### Insecurity

Adolescents and young adult participants described forms of insecurity within their neighborhoods as risk factors for sexual violence, including that sexual crimes often happen in evenings or at night, for example, when girls walk home late from school, or on routes to social events that young women attend:

They look for school girls and they know that those girls come out of school late in the evening, so they hide somewhere during those hours … to threaten the girl. (Group 2, female, rural primary school)

… if a person is sent to the shop at night or a girl might be sent, by bad luck she comes across boys who take her by force and rape her. (Group 6, female, peri-urban primary school)

#### The tourism industry

This industry was perceived by some young people and stakeholders as having a substantial impact on adolescent SRB in Kilifi. The presence of tourists was seen as increasing risks of sexual exploitation of minors locally. In addition, some young people were described as seeking sexual relationships with tourists, based on perceptions of financial benefits that would follow:

… the girls might have more elderly people; the *wazungu* [a term used locally to refer to Caucasian people] …, because they have a good figure, when they go there to the beaches, the *wazungu* get attracted to them then the *wazungu* start (meaning sexual relationships) … because the most people believe that *wazungu* have more money than the locals. (Group 5, male, peri-urban primary school)

#### Social media

Increased access to certain forms of social media and digital communication platforms was considered a predisposing factor for SRB. Some adolescents were thought to have easier access to sexual networks through these channels, especially when they owned mobile phones or had access to social media platforms like Facebook^©^. In this situation, “grooming” was also described as a potential risk:

… in other cases you may meet someone in Facebook. You may become friends in that you arrange for dates. The person may turn not who you expected and he may force you to have sex. (Group 8, female, peri-urban secondary school)

Other participants suggested that SRB is influenced by watching videos and movies with pornographic content.

To a lesser extent, some additional social environmental risk factors were mentioned and these included the following: observing and learning SRB from other community members; also the geographical area of residence was thought to influence SRB although both rural areas and more urbanized areas were described as increasing risk by different participants. Some key informants and young people felt that female adolescents living in the rural part of Kilifi are more likely to face injustices of sexual exploitation like early marriage and coerced sex. However, one key informant also felt that Kilifi town’s fast rate of urbanization can be linked to increase in the various cases of SRB such as prostitution.

### Individual-level factors

#### Low risk awareness

Adolescents were seen to have low awareness of the risks of contracting sexually transmitted infections through unprotected sexual activity, a characteristic associated with SRB. It was shared that, for many sexually active adolescents, the fear of getting pregnant was greater than that of contracting sexually transmitted infections. In this situation, adolescents were seen as more likely to use birth control methods, such as emergency contraceptive pills, that give little or no protection against sexually transmitted infections instead of using condoms. Moreover, adolescents were seen to make judgments in ways that reflected their immediate concerns and interpretations of risk and were not necessarily well informed:

… we got 5 girls who told us … What they use is the morning after pills. They said, “We fear giving birth unlike contracting HIV/AIDS. With HIV/AIDS, we know after getting it, there are ARVs. Life will continue, but with the baby, I think our dreams can be shattered.” (KI, secondary school teacher, female, 32 years)

Some boys may assume that a girl is free of diseases depending on where they come from or who they have slept with, and not use protection. When they have some doubts or they know of a girl’s relative who is sick that is when they may use protection. (Group 3, male, rural secondary school)

However, a few adolescents felt that some adolescents possess a high perception of risk for HIV infection or unwanted pregnancy, and as a result, they may refrain from risky sexual practices like unprotected sex.

#### Coping with and response to biological changes

Many young people perceived that adolescents face challenges in coping with their own physical and psychological developmental changes during adolescence or those of their opposite sex. These may influence their responses, sometimes in ways that predispose them to SRB. In relation to physical changes, some described that the development of secondary sexual characteristics in young women could increase the risks of their becoming targets for sexual harassment. It was thought that some young men may experience a very strong sexual attraction to girls who have developed secondary sexual characteristics in ways that they (the men) find difficult to control:

So the girls might have big breasts and the hips. So when a boy sees that, he might have that feeling to go and have sex. (Group 5, male, peri-urban primary school)

Other biological risk factors for SRB described by participants included the following: (1) age, where older adolescents were seen as more likely to take risks than younger adolescents; and (2) personality characteristics, for example, that being adventurous may involve exploring new experiences that predispose to SRB.

#### Difficulties in negotiating safe sex

Some young people and stakeholders perceived that adolescent girls often do not have control over the way that sexual relations are conducted and, therefore, face challenges in negotiating safe sex. These challenges were seen to result from men’s (who in many cases are older) direct refusal to use condoms or due to manipulation and accusations about unfaithfulness from the male partners:

The girls usually prefer using protection but some of the men are the ones who refuse. Very few men use protection. (Group 6, female, peri-urban secondary school)

#### Conduct and behavioral problems

Some young adults and students perceived conduct and behavioral problems, for example, disobedience and failing to follow parental guidance or teachers’ instructions, as risk factors for SRB among adolescents. Likewise, alcohol and other substance use behavior was also seen as predisposing to SRB.

#### Materialism

Some participants described materialism as an attitude underpinning individual SRB, referencing (as before) that sex may be seen as a form of transaction, allowing adolescents to acquire goods they could not otherwise afford:

So because a boy has promised to buy you heels, a pair of heels and another one has promised to be doing for you all your makeups … every boy will propose to you for as long as he will meet your daily needs. (Group 6, female, peri-urban primary school)

### Family-level factors

#### Family poverty

This was widely perceived to predispose adolescents to SRB. Poverty was described in terms of a failure by caregivers to provide for a young person’s basic needs such as school fees, clothing, food, sanitary pads to adolescent girls, transport fare, and pocket money. As before, this can lead adolescents to engage in transactional sex to acquire these necessities:

Like when we girls are on our periods and you have requested your parents to give you money but they refuse, at that point you really don’t know what to do so you are forced to go out and search from the sugar daddy. (Group 2, female, rural primary school)

Characteristic of family poverty, overcrowding, and poor housing were also linked to a lack of privacy that may lead adolescents to learn about and imitate their parents’ sexual behavior at an early age:

The environment a child is brought up in matters. If they are raised in a small room where the parents sleep with them they get to learn about sex from watching their parents. (Group 1, male, rural primary school)

#### Sex offenders being close family members

Perpetrators of sexual violence were also perceived sometimes to be close family members. These views were shared by young people and stakeholders who described that a number of cases of forced sex, incest, and sexual harassment are perpetrated by family members; most of whom are fathers, step fathers, or male siblings:

Most of the perpetrators (of sexual crimes) … are known people, it can be a cousin, a brother, even can be a father. (KI, medical social worker, male, 27 years)

Such crimes were often thought to be revealed long after they have occurred. In addition, children were seen to be particularly vulnerable to these forms of sexual exploitation since they were dependent on their parents for all their basic needs:

In my view, also parents molest their kids. Also, the kid fears that in case the parent gets caught, she has nobody to provide for her. (Group 4, female, rural secondary school)

#### Parenting

As the preceding paragraphs indicate, the form that parenting takes was perceived as important in both protecting from and in predisposing adolescents in Kilifi to SRB. While some parents’ behaviors could directly put their children at risk of sexual exploitation, both young people and other stakeholders in this study felt that parents’ roles were very important in guiding their children towards behaviors likely to be protective against SRB. Positive parental influences included encouraging sexual abstinence and keeping track of their children’s movements, friendships, and pastimes. As an example of mothers modeling responsible contraceptive practices, a female peri-urban secondary school student explained,

They will get to know about family planning pills from their mothers … so they buy them to avoid pregnancies … they are buying those medicines because their mothers have shown them. (Group 8, female, peri-urban secondary school)

Showing complexity in the way that parent–child relationships could impact SRB, some participants noted that adolescents may be reluctant to utilize certain ASRH services because this could cause tension between them and their parents. In addition, a few participants felt that parents at times may pressurize their children into risky behavioral choices such as early marriage.

### Peer-level factors

#### Peer influence

For many of the risk factors described earlier, peer influence or pressure was perceived as an important additional influence on adolescents’ behavior. Young people explained that some adolescents may have friends who engage in some forms of SRB, and for them to fit into the group, they may engage in such forms of risky behaviors. This influence is coupled with negative peer social norms surrounding the use of contraceptives. These views were also shared by stakeholders:

One was maybe influenced by a friend who practiced these sexual behaviors, then find him/herself trying to practice the same. (Group 4, female, rural secondary school)

Most of the young girls who have no money supply, they usually get influenced by their colleagues to have sex for money. (KI, CBO employee, male, 27 years)

At the same time, peer influence was seen sometimes, if less often, to act in positive ways. As a key informant explained,

… there were 3 youngsters who were around the ages of 15, 13 … then they were talking about how the boy should get condoms and carry the condoms with him if he wants the girl. (KI, secondary school teacher, female, 51 years)

### School-level factors

#### School environment

Young people and stakeholders perceived the school environment as a source of both protective and risk factors for SRB among adolescents in Kilifi. Risks related to the school environment were seen as important potential risk factors. Some adolescents described situations where school teachers have sexually abused students:

There is a child in one of the schools in Kilifi … she was raped by a teacher. That teacher was making her stay behind all the times and then the girl did not know because she was young … she was in class six… (Group 6, female, peri-urban primary school)

Some key informants also described sexual relationships between pupils at school as an important and common risk factor for sexual health. One clinician explained this situation as follows:

… especially when school opens, like in September when … there are burst condoms, unprotected sex and increase in prostitution. (KI, clinician, female, 30)

In connection to protective benefits, some adolescent boys attending primary schools in peri-urban areas felt that enforced rules and regulations would make sure that students focused on their studies and would be less likely to engage in SRB. School attendance itself was perceived by young people as a protective factor, through enhancing ASRH knowledge and prompting safer sexual behavior. Additionally, some stakeholders felt that specific school structures or programs with an ASRH aspect offered significant protection against SRB, including school clubs and school counseling and guidance services. As one school teacher explained,

You see also in our school, guidance and counseling is being emphasized. … we are encouraging them, we have this club of child rights. And we also have some signs which we are looking at from the girls. It is easy to identify that such a girl is undergoing torture; may be sexual abuse, physically, psychologically. (KI, primary school teacher, male, 48 years)

#### School dropout

Furthermore, dropping out of school was discussed in a few adolescent FGDs as contributing to sexual risk-taking. For this group, the difficulty in finding paid employment typical of the area could lead adolescents to take on transactional forms of sex to obtain gifts or money.

### Macro-level factors

A few participants (including two teachers, two CBO employees, and a clinician) discussed macro-level risk factors, that is, factors that operate at a population level.

#### Societal changes

Among the macro-level factors noted was the perception that a set of changes in society over time have together promoted risky sexual behavior. These changes included that adolescents normalize early sexual behavior, a deteriorating quality of parenting, and a general sense that society has lost its moral basis. As one key informant shared,

I can go to a rural place there, and am sure this lady has a boyfriend or is married, but if I have money I can just take the lady from him … that is the problem with the society now days. It is about you being financially stable. It is not about the traditions and morals anymore. (KI, CBO employee, male, 27 years)

#### Restrictive ASRH policies

Also, some policies surrounding ASRH were considered restrictive. Some stakeholders explained that although there is demand by adolescents for certain ASRH services, like longer term contraception, the present policy framework prohibits provision of such services to those under 18 years:

This injection that women get. In fact it is the most preferred contraception among the girls … the private doctors will give them. It is an illegal thing but you realize that most of the girls will be using it. (KI, secondary school teacher, female, 51 years)

Another participant who is a clinician also stated,

… the issue was with the policies. We couldn’t give contraceptives to those under 18. (KI, clinician, female, 30 years)

#### Traditional customs and beliefs

Some key informants suggested that certain traditional beliefs and customs, particularly those encouraging early marriage, may infringe adolescents’ rights and freedom of choice, as well as predispose to SRB. As described earlier, this issue also acts at the level of the family, highlighting diversity between family contexts and the interrelated nature of categories within the Ecological Framework drawn on in this article:

One which is common is the old men taking young girls. In our culture that is very common. The Mijikenda men, they believe that after a certain age such as 40 and above, a man should have a younger wife. We call such a wife Kiboma. (KI, primary school teacher, male, 48 years)

#### Legal and punitive action

Some young people and key informants shared the views that presence of legal and punitive action for sexual crime perpetrators partly helps to minimize sexual-related violence and other forms of SRB. They gave various scenarios in which the local authorities such as the local police and Chief’s office took action when such crimes were reported in Kilifi. One, for example, narrated,

Somebody sleeps with his own daughter … until she becomes pregnant, the case is taken to the Chief … There is one who was imprisoned just the day before yesterday. (Group 11, male, young adult)

## Discussion

Young people and stakeholders’ perceptions suggest that a range of SRBs occur among adolescents in Kilifi, especially transactional sex, early sexual debut, unprotected sex, and multiple sexual partnerships. Similarly, sexual violence is reported as common and the adolescent girls seem to bear the brunt. The occurrence of SRB and violence among Kenyan adolescents (Juma et al., 2014; Marston et al., 2013) and adolescents from other parts of SSA (Moore et al., 2007) is widely documented. Our findings support those of reports from the Kenya Demographic and Health Survey (KNBS and ICF International, 2014) that show a high occurrence of teenage pregnancies and payment for sex in Kilifi County, although this health survey data did not comprehensively explore factors underlying these behavioral patterns.

Our findings also indicate that the social environment presents a milieu for a majority of the risk factors. Significant among these factors is the lack of pragmatic youth-friendly ASRH services. We suggest that the difficulties in safe sex negotiation, and insufficient knowledge and misconceptions about sexual health described by participants in this study are likely consequences of poor ASRH services. Similar to our findings, socio-psychological factors such as concerns about confidentiality, fear and embarrassment, and poor accessibility and unaffordability have been described as serious bottlenecks for youth-friendly services targeting adolescents from Kenya (Godia et al., 2014; National Coordinating Agency for Population and Development (NCAPD) et al., 2011) and across SSA (Biddlecom et al., 2007; Geary et al., 2014). With only 7 percent of Kenyan health facilities providing youth-friendly services (NCAPD et al., 2011), the need for comprehensive adolescent-friendly ASRH remains urgent.

Furthermore, our participants’ views underscore the devastating impacts of household poverty on ASRH. These effects are cross-cutting and may link to certain social environment factors like engagement of adolescent girls in risky commercial activities, dropping out of school, and sexual exploitation of adolescents by tourists following the pursuit for financial and material gains. Likewise, poverty may propagate materialistic tendency portrayed by some adolescents as earlier reported under the individual-level factors. Indeed, survey data from across Kenya show that adolescents from the most poor households are most likely to be engaged in sexual activity and not to be using contraceptives both in rural and urban areas (KNBS and ICF International, 2014). Although this is the case, the evidence is mixed in that some studies indicate that household income may or may not predict certain forms of SRB and that these effects may vary from one country to another, or by sex and age of the adolescent (Berhan and Berhan, 2014; Handa et al., 2016; Madise et al., 2007) This points to a need for more research to delineate the effects of household income on adolescent sexual health.

Additionally, parenting style was another topic of discussion with views that “neglectful” parenting predisposes adolescents to SRB and that there are opportunities for parents to support safer behavior in relation to sexual health. We propose that this factor is linked to other perceived risk factors such as negative modeling, school dropout, and attendance at “risky” social events. Correspondingly, a study from rural Western Kenya identified poor caregiver monitoring as a predisposing factor for SRB among adolescents (Puffer et al., 2011). To this end, findings from other parts of SSA also indicate that low levels of parental monitoring and poor parent–adolescent communication are associated with elevated risk of adolescent sexual activity for both male and female adolescents (Biddlecom et al., 2009). This highlights the need for SRH interventions to incorporate aspects of parental or caregiver involvement.

The current findings also suggest that insecurity within the community facilitates the occurrence of various forms of sexual violence, especially against adolescent girls. More worrisome is that home and school environments where the majority of the adolescents spend most of their time are perceived as unsafe. These views are consistent with other findings from Kenya, which indicate that home environment may often be unsafe for adolescents and that perpetrators of sexual crimes are most likely to be close family members such as fathers and siblings, or peers and teachers at school (Ruto, 2009; UNICEF et al., 2012).

Emerging from these issues, our findings highlight that gender inequality is a major concern in relation to adolescent SRB and human rights more broadly. In our data, varying risk factors associated with gender cross ecological domains to reinforce susceptibility to SRB, especially for the female adolescents. Among these risk factors are the Mijikenda patrilineal culture that supports male dominance, thereby creating power asymmetries at environmental and family levels; poverty that disproportionately exposes girls and young women to sexual harms and exploitation; and gendered cultural practices such as early girl marriage. Gender inequality has also been highlighted in other studies from Kilifi with examples such as maternal blame for health problems within the family and the lack of autonomy for females to participate in certain health programs without permission from men within the household (Kamuya et al., 2015; Marsh et al., 2011). These findings emphasize the need to incorporate an intersectional perspective (Shields, 2008) in delineating the cross-cutting influence of gender for risk factors surrounding ASRH. This is important because differences in individual’s control over resources and power are often embedded within social identities such as gender, age, and ethnicity (Shields, 2008). These power dynamics can generate unfair or negative beliefs, attitudes, and values surrounding sexual behavior.

Our findings also underscore the enormous impact of negative cultural beliefs, norms, and customs on adolescents’ SRB. Their views indicate that some existing customs and beliefs such as early marriages infringe on adolescents’ rights and freedom while others practices during events like burial ceremonies predispose them to high-risk experiences. These experiences concur with findings from other parts of Kenya (Juma et al., 2014; Njue et al., 2009), as well as SSA (Boyden et al., 2012; Wadesango et al., 2011), where such practices have been linked to poor health and social livelihood. Thus, ASRH interventions need to consider the specific socio-cultural dynamics that impact adolescents’ sexual health.

Concerning protective factors, few opportunities were discussed by participants and this may reflect the poor state of ASRH services in this region or a general lack of awareness of available services and structures for ASRH. Nonetheless, some participants acknowledged the existence of certain protective factors, especially school-based ASRH services and structures like teen clubs, guidance and counseling teachers, and functional school regulations. Indeed, school attendance has been attributed with large protective effects for early sexual debut among Kenyan adolescents (Handa et al., 2016; KNBS and ICF International, 2014), which emphasizes the need to enable school attendance and strengthen existing school structures by incorporating opportunities for ASRH promotion. There is a need to identify and strengthen both formal and informal systems that can be protective and promotive factors for ASRH in our context and similar contexts.

Besides, much as ASRH services are not yet comprehensive or adolescent-friendly (NCAPD et al., 2011), access to some form of ASRH services remains an important protective factor. These findings indicate that there is a mix of private, non-profit organization, and public service providers offering a range of ASRH services like short- and long-term contraception methods. While some of these services are free or subsidized for adolescents, more needs to be done in making the relevant ASRH services more usable, accessible, and affordable for adolescents. These are also major issues that adolescents grapple with from other parts of SSA and the rest of the world and have been earmarked for much-needed intervention (Bearinger et al., 2007).

These findings highlight the perceived importance of legal or punitive action for sexual offenders in handling and addressing sexual violence and injustices against adolescents in Kilifi. Indeed, the Kenyan laws on sexual offenses have been recently amended (National Council for Law Reporting (NCLR), 2014; Ndungu, 2007) to extensively define sexual violence beyond mere focus on morality. New themes that had not been clearly addressed in the older laws include criminalization of sexual harassment, child sex tourism, child pornography, gang rape, incest, and child prostitution (NCLR, 2014). There are also provisions for the protection of vulnerable witnesses, entitlement of both victims and perpetrators to treatment and professional counseling, and long-term supervision of dangerous sexual offenders after serving their prison sentences (NCLR, 2014). Notwithstanding, sexual crime is still problematic in Kilifi (NCRC, 2014), and this may partly be tied to not only low levels of awareness by the public about their rights and existing laws on sexual crime but also low levels of awareness by key stakeholders such as police and public administrators who are in charge of implementing these laws (Ndungu, 2007). Overall, while evidence surrounding the effectiveness of legal action for sexual perpetrators remains scanty, especially in the SSA context, restorative justice or human rights approaches for handling sex offenders are clearly important (McAlinden, 2005; Ward et al., 2007).

Overall, this study has several strengths. Our participants were a diverse mix of adolescent sub-groups, that is, primary and secondary school adolescents, those who had dropped out of school and adolescents living with HIV. They were also recruited with a representation of both rural and peri-urban settings in Kilifi County. In addition, findings were triangulated across the different methods used to consider those from FGDs and key informants. At the same time, in interpreting our findings, it is important to take account of limitations for this study. First, the views shared by our participants may be specific to adolescents living in Kilifi at the Kenyan coast. Although similar views about SRB may be shared from other geographical contexts, we are not in position to ascertain the relevance of these findings to other groups or populations. Additionally, as for qualitative methods in general, data collection, analysis, and interpretation are subject to individual influence; we have countered this effect through maintaining reflexivity and ensuring the active contribution of a multi-disciplinary research team in all stages of this research.

## Conclusion

Overall, our findings stress that SRB is a significant problem among adolescents at the Kenyan coast. Many of our findings highlight the generalizability of risk factors that have been documented in studies in other regions of Kenya and beyond, particularly the gendered nature of such risks. However, novel risk factors in the Kenyan coastal context include the following: the negative impact of the tourism industry on adolescents’ sexual behavior, the severe implications of family poverty such as engagement of adolescent girls in risky commercial activities, and the negative consequences of certain customary events like funeral ceremonies. Moreover, these factors coexist or interplay across different ecological levels. Important measures to counter SRB in this and similar contexts are likely to include the following: useful and accessible ASRH services for adolescents; improved safety within the adolescents’ neighborhood, home, and school environments; responsible parenting; tackling oppressive and potentially harmful cultural norms and customs; ensuring that legal and other regulatory processes are recognized and enacted in these settings; and addressing poverty within households. Gender inequality remains a major concern that should be explored and addressed with special attention to the intersectionality of its aspects across ecological domains. Opportunities for improving ASRH exist. First, many Kenyan adolescents are school goers and thus could be accessed with a range of ASRH services through the education system. Second, a presence of diverse private, public, and civil society service providers in the region presents existing platforms for resource mobilization and through which improvement in access plus quality of ASRH can be realized. Third, a comprehensive legal framework and national guidelines for management of sexual violence in Kenya exist and thus need more public awareness and more effective implementation. To this end, intervention research should focus on delineating effective program and policy action to tackle these drivers of adolescent SRB at different ecological levels in Kilifi and other geographical settings.
